# Heparan sulfate functions are altered in the osteoarthritic cartilage

**DOI:** 10.1186/s13075-020-02352-3

**Published:** 2020-12-07

**Authors:** Sara Shamdani, Sandrine Chantepie, Camille Flageollet, Nadia Henni-Chebra, Yohann Jouan, Florent Eymard, Eric Hay, Martine Cohen-Solal, Dulce Papy-Garcia, Xavier Chevalier, Patricia Albanese

**Affiliations:** 1 Univ Paris Est Creteil, Gly-CRRET, Glycobiology Cell Growth Tissue Repair and Regeneration Research Unit, Créteil, F-94010 France; 2 INSERM, UMR-S 1132 Bioscar, Centre Viggo Petersen, Hôpital Lariboisière, 2, Rue Ambroise Paré,, Creteil, F-94010 France; 3 BIOSCAR Inserm U1132, Université de Paris, F-75010 Paris, France; 4 INSERM, UMR-S 1132 Bioscar, Centre Viggo Petersen, Hôpital Lariboisière, 2, Rue Ambroise Paré, Créteil, 75010 France; 5 Université de Paris (UFR de Médecine), Paris, 75010 France

**Keywords:** Heparan sulfate, Sulfation, Osteoarthritis, Cartilage matrix, Chondrocytes

## Abstract

**Background:**

Heparan sulfate (HS) proteoglycans (PG) may be found at the chondrocyte surface and in the pericellular cartilage matrix, and are involved in cell-cell and cell-matrix interactions. An important function of HS chains is to regulate cell fate through specific interactions with heparin-binding proteins (HBP) modulated by their complex sulfation pattern. Osteoarthritis (OA) is a joint disorder characterized by the degradation of articular cartilaginous extracellular matrix. The aim of this study was to investigate HS structure and functions in osteoarthritic cartilages compared to normal cartilages (controls).

**Methods:**

Glycosaminoglycans (GAG) were extracted from human macroscopically normal cartilages (controls, *n* = 7) and (OA cartilages *n* = 11). HS were isolated and quantified using the DMMB quantification method. Their structure and functions were then compared using respectively a HPLC analysis and HBP binding tests and their phenotypic effects on murine chondrocytes were studied by RQ-PCR. Statistical analyzes were performed using a one-way ANOVA followed by a Dunnett’s test or a *t* test for pairwise comparisons.

**Results:**

In OA, HS were characterized by increased sulfation levels compared to controls. Moreover, the capacity of these HS to bind HBP involved in the OA pathophysiological process such as FGF2 and VEGF was reduced. Chondroitin sulfates and keratan sulfates regulated these binding properties. Finally, HS from OA cartilages induced the mRNA levels of catabolic markers such as MMP3, MMP13, and TS4 and inhibited the mRNA levels of anabolic markers such as COL2, ACAN, SOX9, and VEGF in murine articular chondrocytes.

**Conclusion:**

The sulfation of HS chains was increased in OA cartilages with changes in HBP binding properties and biological effects on chondrocyte phenotypes. Thus, modified HS present in altered cartilages could be a novel therapeutic target in OA.

## Background

Osteoarthritis (OA) is the most common disabling joint disorder and is associated with a high economic burden. It is characterized by joint cartilage degradation, subchondral bone remodeling, and synovitis [1]. A damaged articular cartilage has a poor capacity for self-repair and there is so far no efficient curative treatment available for OA [2]. The articular cartilage performs biomechanical functions within the joint, sustained by an extracellular matrix (ECM) very rich in fibrillar proteins such as collagens, and in proteoglycans such as aggrecan. As a result of ECM injuries or changes in ECM quality with aging, chondrocytes become hyper-activated. They secrete different degrading matrix enzymes and decrease anabolic activities, triggering their own hypertrophy and the mineralization process, that are characteristic stages of OA [3]. Among ECM components, glycosaminoglycans (GAG) are long anionic polysaccharides that form highly complex structures subdivided into four subfamilies according to their repeated disaccharide building blocks: heparan sulfates (HS), chondroitin sulfates (CS), keratan sulfates (KS), and hyaluronic acid (HA). The structural complexity of GAG is due to the heterogeneity of their disaccharide composition, their glycosidic linkages, and their various sulfation patterns. Sulfated GAG (HS, CS, and KS) are covalently bound to core proteins to form proteoglycans (PG), whereas non-sulfated HA remains free in the ECM.

Studies assessing cartilage ECM remodeling in aging and OA have mainly been focused on the main PG called aggrecan and on the structural and moisturizing functions of its CS and KS chains [4–6]. Indeed, a reduced size in and the loss of sulfated CS and KS chains alter the integrity and biomechanical properties of the cartilage matrix [4, 5]. However, heparan sulfate proteoglycans (HSPG), including the syndecan and glypican families, are also present in the cartilage, but at lower concentrations [7]. HSPG are involved in the functional regulation of cell properties through their capacity to specifically interact with heparin-binding proteins (HBP), including growth and/or differentiation factors, cytokines, chemokines, morphogens, and enzymes [8]. This is mainly due to the complex structures of HS that vary in terms of sulfate and acetate group contents, and length of their sulfated domains. HS protect HBP from their proteolytic degradation and trigger their activities. They also provide matrix storage sites for HBP prior to their binding to cell surface components [9, 10]. Therefore, fine alterations of HS structures affect their ability to regulate the activity of trophic factors on cell functions, leading to a potential loss of tissue function, as previously shown in aging [11, 12] or in various diseases [13, 14]. Due to their low concentrations and to the difficulty in purifying them, only a few studies have assessed HS structural evolution and their biological functions in the cartilage. The aim of this study was thus to assess HS structure and functions in OA cartilages compared to control cartilages.

## Methods

### Human cartilage samples and clinical characteristics

Cartilage explants were obtained from human subjects. All subjects provided their informed consent and a legal authorization was provided by the Ethics Committee of the Rheumatology Department of Henri Mondor Hospital (no. 07-34). Demographics and clinical data were collected (Table 1). Explants were taken from the knee of OA patients (*n* = 11) undergoing total knee replacement surgery. These OA explants originated from the femur (F), tibial plateau (TP), and patella (P) were sampled 1–4 h after ablation from the joint. Control explants (*n* = 7) were taken from a macroscopically normal cartilage part of the femoral head of control subjects after neck fracture. The extent of wear of all explants was assessed using the Kellgren-Lawrence grading system (0–4) [15], and explants were then immediately frozen at − 80 °C until used. Patients were not involved in the design, conduct, reporting, and dissemination of the research.

**Table 1:** Patient’s clinical characteristics. Data is expressed as the mean ± SEM of values from healthy control donors (CT) and osteoarthritis patients (OA) according to the gender (female (F), male (M)) and for all subjects

Patients	CT			OA		
**Sex**	**F**	**M**	**All**	**F**	**M**	**All**
Number	6	1	7	5	6	11
Age (year)	82 ± 5	88	83 ± 4	78 ± 9	74 ± 6	75 ± 7
Lenght (cm)	160 ± 1	178	166 ± 8	156 ± 2	178 ± 3	169 ± 10
Weight (kg)	56 ± 6	95	64 ± 12	66 ± 2	97 ± 16	84 ± 19
**OA severity**						
(K-L global)	1.7 ± 0.4	1	1.6 ± 0.5	4 ± 0	4 ± 0	4 ± 0
Menopause	Yes	NC		Yes	NC	
Analgesics	NC	NC	NC	0	3	3
AINS	NC	NC	NC	0	1	1
Corticoid	NC	NC	NC	1	0	1
Hyaluronic Acid	NC	NC	NC	1	0	1
Period of symptoms (Year)	NC	NC	NC	12.5 ± 2.5	9 ± 3	10 ± 2

OA severity was assessed using the Kellgren-Lawrence (KL) radiographic classification of knee for OA: 0 = normal, 1 = doubtful, 2 = minimal, 3 = moderate and 4 = severe. *NC* not concerned, *NSAID* non-steroidal anti-inflammatory drugs

### Extraction and quantification of GAG from articular cartilages

GAG were extracted as previously described [11]. Briefly, freeze-dried samples were weighted, homogenized and suspended in digestion buffer (50 mM Tris-HCl, 10 mM NaCl, 3 mM MgCl_2_, 1% Triton X-100, pH 7.9) to a final concentration of 50 mg of tissue per mL. Samples were incubated with proteinase K (Sigma-Aldrich, final concentration 100 μg/mL) at 56 °C for 24 h, followed by enzyme inactivation at 90 °C for 30 min. DNA was then digested with DNase I (Qiagen, 10 U/mL of sample) overnight at 37 °C. Samples were filtered (PALL life science ODM02C34) and centrifuged (12,000 rpm, 10 min, at 6 °C). NaCl was added to the filtrates at a final concentration of 4 M and samples were stirred for 30 min at room temperature (RT). Samples were incubated with 10% TCA (Sigma-Aldrich), cooled at 4 °C for 15 min, and centrifuged (12,000 rpm, 10 min, 4 °C) and the supernatants were recovered. Lipids were eliminated by chloroform extraction (1:1) and centrifugation (12,000 rpm, 10 min, 8 °C). The aqueous fractions were dialyzed (Thermo scientific 3.500 MWCO) against a buffer (50 mM Tris, 50 mM CH3COO-Na, 2 mM CaCl_2_, pH 7) and then against pure H_2_O. Samples were frozen at − 80 °C and freeze-dried. Finally, samples were re-suspended in pure H_2_O to a desired tissue weight/H_2_O volume concentration and stored at − 20 °C until use. Extraction/quantification quality controls (QC) were included in each extraction process, by spiking a known amount of GAG from some freeze-dried cartilage samples to calculate the GAG extraction yield.

To isolate GAG species, total GAG samples were diluted in glycanase digestion buffer. To isolate HS, keratanase (2 mU/mL; Amsbio PS170615) was added for 1 h at 37 °C, followed by chondroitinase ABC (300 mU/mL, SIGMA C3667) for 1 h and 30 min at 37 °C. To isolate CS, samples were incubated with keratanase and then a mix of heparitinases I, II, and III (250 mU/mL for each, Iduron) was added overnight at 37 °C. To isolate KS, samples were incubated with chondroitinase ABC (300 mU/mL, SIGMA C3667) for 1 h and 30 min at 37 °C and then a mix of heparitinases I, II, and III (250 mU/mL for each, Iduron) was added overnight at 37 °C.

### Dimethyl-methylene blue (DMMB) assay for quantification of sulfated GAG

Sulfated GAG were quantified using the 1–9 dimethyl-methylene blue (DMMB, Sigma-Aldrich) assay as previously described [16]. CS, KS, and HS were quantified in each sample after enzymatic and/or chemical reactions. QC for total digestion and GAG specificity were included by spiking samples with known amount of commercial CS, KS, or HS. CS were quantified after digestion with chondroitinase ABC (Sigma-Aldrich) as previously described [16]. HS were quantified after incubation with nitrous acid as previously described [16]. KS were quantified according to 2 strategies. First, HS and CS were digested with chondroitinase ABC (300 mU/mL) and a mix of heparitinases I, II, and III (Iduron, 250 mU/ml for each) for 1 h at 37 °C. The remaining GAG corresponding to KS were quantified according to the DMMB protocol. In a second strategy, samples were incubated with keratanase (2 mU/mL; Amsbio) for 1 h at 37 °C and the remaining GAG (HS+CS) were quantified according to the DMMB protocol using a KS calibration curve. The amount of KS was calculated as the difference between the amount of total GAG and the amount of the remaining GAG (HS and CS) quantified in a given sample. The values given per sample are the mean of the values obtained with both strategies.

### Structure of HS and CS determined by HPLC

The overall sulfation pattern of HS and CS disaccharides was determined by HPLC as previously described [11]. Briefly for CS analysis, CS disaccharides were obtained by sample digestion with chondroitinase ABC (300 mU/mL, Sigma-Aldrich) for 1 h and 30 min at 37 °C. For HS analysis, due to a high amount of CS in the samples, a double digestion of the total GAG extracts was performed with digestion with chondroitinase ABC (300 mU/mL, Sigma-Aldrich) for 1 h and 30 min at 37 °C and then incubation with a mix of Heparitinases I, II, and III (Iduron, 0.25 mU for each) overnight at 37 °C. The HS disaccharide composition was determined by subtracting the signal of CS peaks previously obtained from (HS+CS) peaks. Digested samples (CS and HS+CS disaccharides) were filtered, loaded onto a HPLC Proteomix SAX-NP5 column, and eluted with a solvent gradient as previously described [11]. Results are expressed as a percentage of the area of each peak relative to the sum area of all peaks for each sample.

### Heparin/glycosaminoglycan ELISA competition assay to determine GAG capacity to bind HBP

The capacity of GAG to bind HBP (rh Basic FGF2, rh VEGF-165 (PROMOKINE)) was assessed by an ELISA-based competition assay as previously described [17]. Briefly, 96-well ELISA plates were coated with heparin-BSA conjugates, washed, and saturated with 1X PBS, 3% BSA. Then, HBP were added to each well (FGF2 at 2.5 ng/well; VEGF-165 at 5 ng/well) together with increasing concentrations of GAG extracted from the samples (serial dilutions from 0.01 to 10 μg/mL in PBS) in duplicate. Competition for HBP binding between immobilized heparin and soluble GAG was performed for 1 h in the plate. After washing, HBP bound to heparin coated onto the plate were incubated with a specific antibody (anti-FGF2 Mouse Monoclonal IgG diluted 1/2000, R&D systems MAB233; anti-VEGF Rabbit Polyclonal IgG, diluted 1/500 PROMOKINE PK-AB815-64420) for 1 h at RT and then with a HRP-conjugated secondary antibody (HRP-AffiniPure Donkey Anti-Mouse IgG diluted 1/5000, Jackson ImmunoResearch 715-035-151; HRP-AffiniPure Goat Anti-Rabbit IgG, diluted 1/2000; Jackson ImmunoResearch 111-035-144). The peroxidase activity was measured by oxidation of the 3,3′,5,5′-tetramethylbenzidine (TMB, Thermo Scientific) substrate. The maximum binding (100%) was determined in the presence of HBP and in the absence of extracted GAG. The IC_50_ was defined as the GAG concentration (μg/mL) inhibiting 50% of HPB binding to immobilized heparin. The IC_50_ was used to calculate relative binding affinities.

### Murine primary chondrocyte cultures and RQ-PCR analysis

Murine articular chondrocytes were taken from 6-day-old mice as described before [18], and then seeded into plates for proliferation. At day 6, primary chondrocytes were incubated without and with 2.5 μg/mL of GAG extracted from cartilages or with recombinant mouse interleukin-1β (IL-1β, R&D systems, 1 ng/mL) for 24 h. Then, the chondrocyte RNA was recovered using Qiazol reagent (QIAGEN). Reverse transcription was performed using the high capacity cDNA reverse transcription Kit (Applied Biosystems). Real-time PCR was performed using SYBR Green Master Mix (Applied Biosystems) to analyze the pattern of mRNA expression of anabolic (COL2, ACAN, SOX9), catabolic (MMP-3 and 13, ADAMTS 4 and 5), and hypertrophic (VEGF) markers, using previously validated primer sequences [19]. Averaged threshold cycle (Ct) values were normalized to the averaged Ct value of the housekeeping gene, HPRT1. Adjusted average Ct values were used to calculate the expression level of each gene relative to HPRT1 expression level.

### Statistical analysis

Each GAG sample was tested in duplicate in 3 independent experiments. The mean of the duplicate was the value of the GAG sample reported for one experiment. Statistical analyzes were performed using Graph Pad Prism 5 software. The statistical significance of differences between groups was determined using an ordinary one-way ANOVA and pairwise comparisons were made using a two-tailed unpaired Student’s *t* test or Dunnett’s multiple comparison test. Data are expressed as the mean ± SEM. A significant *P* value was defined as follows: *< 0.05; **< 0.01, ***< 0.001, ****< 0.0001.

## Results

### Donors

There were 11 OA donors (6 women with a median age of 78 years and 5 men with a median age of 74 years) and 7 control donors (6 women with a median age of 82 years and 1 man aged 88 years). The mean Kellgren-Lawrence score was 1.6 ± 0.5 in the control group and 4 ± 0 in the OA group.

### Sulfated GAG levels are decreased in OA cartilages

**Fig. 1:**
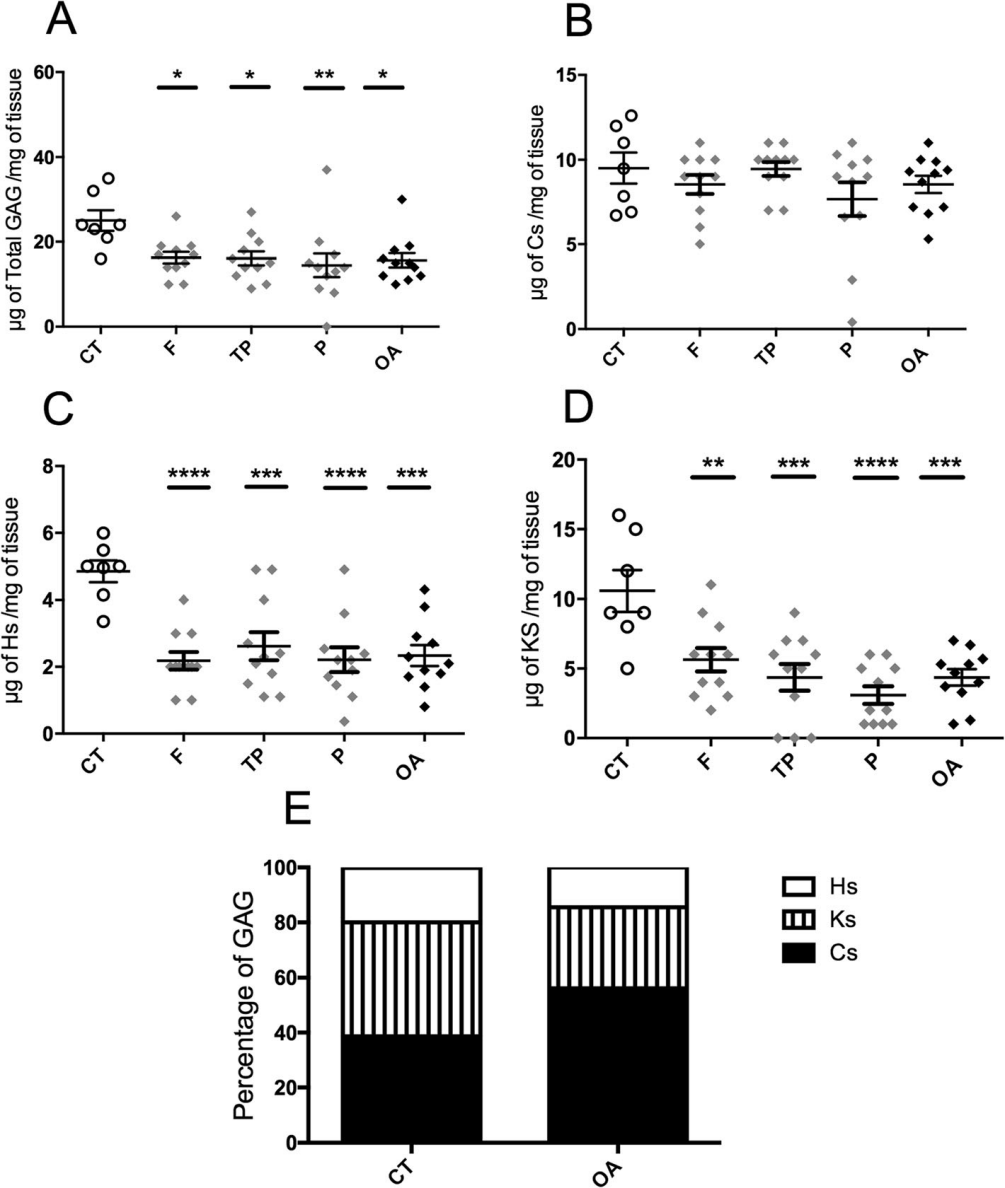
Quantification of sulfated GAG. The amounts of total sulfated GAG (**a**) and isolated CS (**b**), HS (**c**), and KS (**d**) were normalized in μg of GAG per mg of articular cartilage from healthy control donors (CT, *n* = 7) and osteoarthritis patients (OA, *n* = 11). The values of the CT group were compared to the values obtained for the femur (F), tibial plateau (TP), and patella (P) samples from OA patients and to the mean of the 3 samples from each OA patient (OA). The value of a GAG sample was the mean of 3 values obtained from 3 independent experiments. All values per group are expressed as a mean ± SEM. *P* values were calculated using an ordinary one-way ANOVA test followed by pairwise comparisons using the Dunnett test: *< 0.05, **< 0.01, ***< 0.001, ****< 0.0001. **e** Distribution of HS, KS, and CS amounts as a percentage of the amount of total GAG (100%)

The level of total sulfated GAG (Fig. 1a) was significantly lower in OA cartilages (15.6 ± 1.6 μg/mg) compared to control cartilages (25 ± 2.4 μg/mg). Lower CS levels (Fig. 1b) were observed in OA samples (8.5 ± 0.5 μg/mg) compared to control samples (9.5 ± 0.9 μg/mg), without reaching significance. However, significantly lower KS (Fig. 1d) and HS (Fig. 1c) levels were measured in OA samples (4.3 ± 0.5 μg/mg for KS and 2.3 ± 0.3 μg/mg for HS) compared to control samples (10.5 ± 1.4 μg/mg for KS and 4.8 ± 0.3 μg/mg for HS). Based on these overall differences between control and OA samples, the analysis of the percentage of each GAG species relative to the total amount (Panel E) showed that the CS fraction was increased and the most represented in OA cartilages compared to control ones (respectively 56% and 38%). Conversely, the HS and KS fractions were decreased in OA samples compared to control samples (respectively, 20% and 42% of total GAG in control samples versus 14% and 29% in OA samples).

### Sulfated forms of CS and HS disaccharides are increased in OA cartilages

**Fig. 2:**
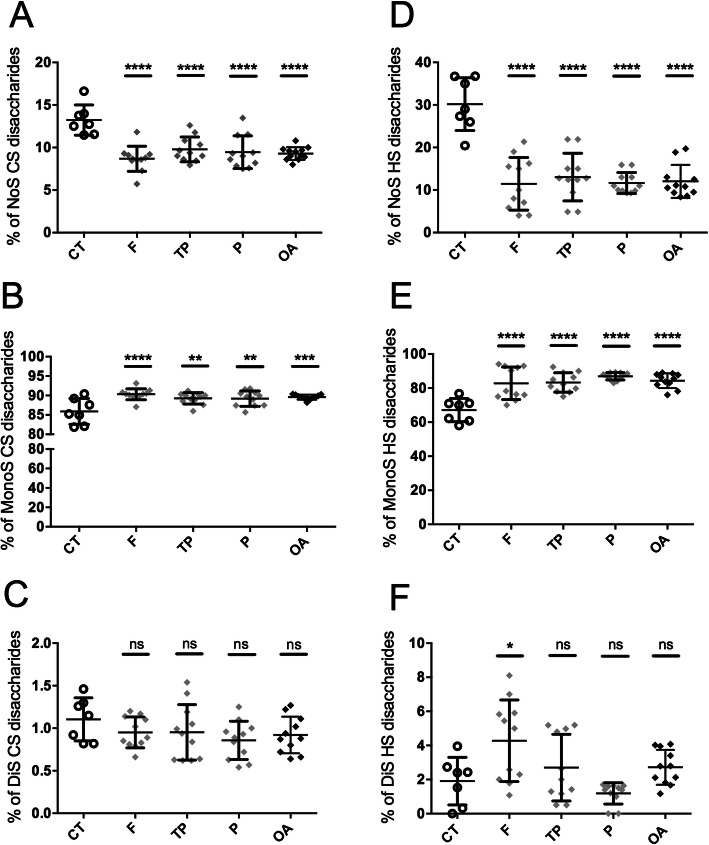
HPLC analysis of the sulfation pattern of GAG disaccharides. A HPLC analysis was performed on the CS (**a**–**c**) and HS (**d**–**f**) disaccharide units contained in the GAG chains isolated from cartilages of CT donors (*n* = 7) and OA patients (*n* = 11), to determine the relative composition of each disaccharide unit: non-sulfated (NoS) (**a**, **d**), monosulfated (MonoS) (**b**, **e**), and disulfated (DiS) (**c**, **f**). Each sample was analyzed twice independently. The value of a GAG sample was the mean of 3 values obtained from 3 independent experiments. All values per group are expressed as a mean ± SEM. The values of the CT group were compared to the value obtained for the different parts of OA cartilages, i.e., the femur (F), tibial plateau (TP), and patella (P), and to the mean of the 3 parts from each OA patient (OA). *P* values were calculated using an ordinary one-way ANOVA test followed by pairwise comparisons using the Dunnett test: *< 0.05, **< 0.01, ***< 0.001, ****< 0.0001

The HPLC analysis showed that CS disaccharides (Fig. 2a–c) were mainly monosulfated in control cartilages (85 ± 1.2%) (Fig. 2b) and that the level of monosulfated CS disaccharides was significantly increased in OA cartilages (89 ± 0.1%). Conversely, the lower fractions of non-sulfated (Fig. 2a) and disulfated (Fig. 2c) CS disaccharides were decreased in OA cartilages (9.2 ± 0.2% and 0.9 ± 0.06%, respectively) compared to control samples (13 ± 0.6% and 1.1 ± 0.09%, respectively). HS disaccharides (Fig. 2d–f) were mainly monosulfated in control cartilages (67 ± 2.5%) (Fig. 2e) and the level of monosulfated HS disaccharides was significantly increased in OA cartilages (84.3 ± 1.2%). This was associated with a 3 times lower non-sulfated HS disaccharide level in OA cartilages (12 ± 1.1%) compared to control cartilages (30 ± 2.3%) (Fig. 2d).

### Changes in the binding affinity of GAG for growth factors in OA cartilages

**Fig. 3:**
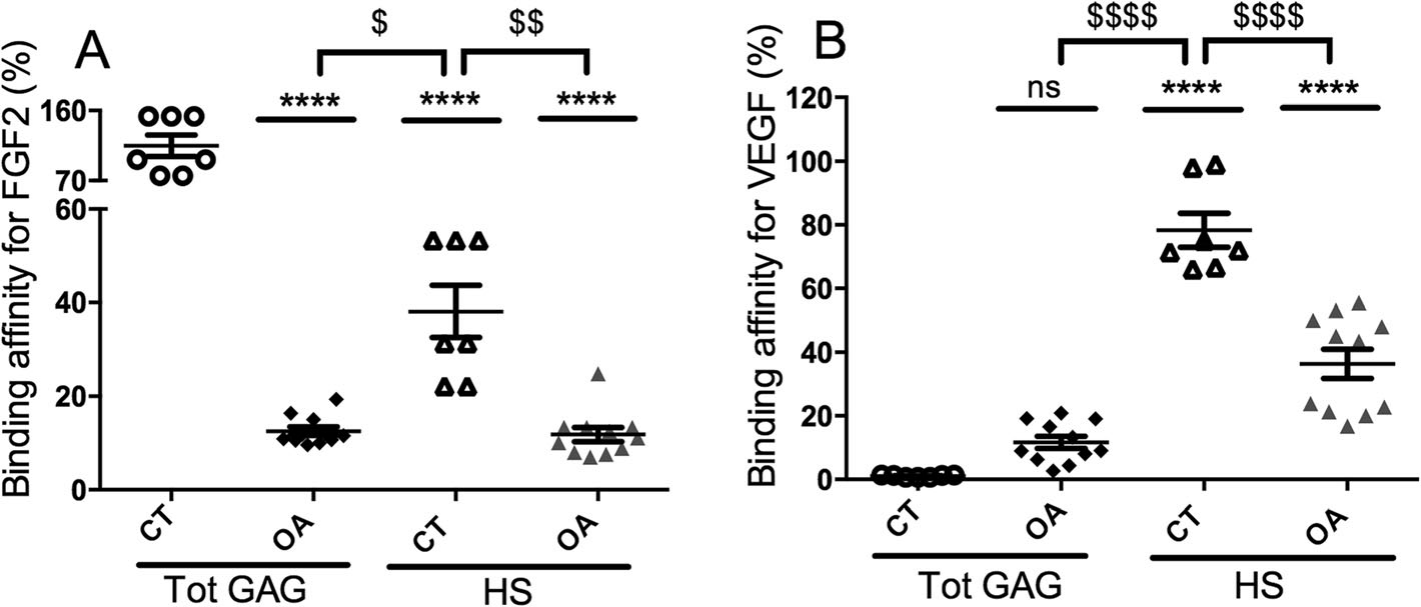
Percentage of binding affinity of total GAG and HS for FGF2 and VEGF. **a** The IC_50_ of total GAG from control articular cartilages (CT, *n* = 7) was considered 100% of binding to FGF2 and used to calculate the % of binding affinity of all other GAG (OA total GAG, *n* = 11, CT HS and OA HS) for FGF2. **b** The IC_50_ of total GAG from control articular cartilages was considered 1% of binding to VEGF and used to calculate the % of binding affinity of all other GAG (OA total GAG, *n* = 11, CT HS and OA HS) for VEGF. Each GAG sample was tested in duplicate in each experiment and the mean of the duplicate was considered the value of the GAG sample. All values per group are expressed as a mean ± SEM. *P* values were calculated using an ordinary one-way ANOVA test followed by pairwise comparisons using the Dunnett test compared to CT total GAG (**< 0.01, ***< 0.001, ****< 0.0001) or to CT HS (^$^< 0.05, ^$$^< 0.01, ^$$$^< 0.001, ^$$$$^< 0.0001)

An ELISA-based competition binding assay (Fig. 3 and Table 2) was used to investigate whether the structural changes in GAG from OA cartilages were associated with a different capacity to bind to HBP that are essential for cartilage homeostasis such as FGF2 and VEGF [20, 21]. Total GAG isolated from control cartilages bound to FGF2 (panel A) and inhibited by 50% its binding to heparin with an IC_50_ of 11 ± 4 μg/mL (Table 2). The value obtained with total GAG from control cartilages was defined as a relative binding affinity of 100% (Fig. 3a). The IC_50_ of total GAG isolated from OA samples (84 ± 20 μg/mL) was 8 times higher (Table 2), corresponding to an 8-fold reduction in the binding affinity of GAG from OA samples for FGF2 (12.5 ± 0.9%) compared to control cartilages (100%) (Fig. 3a). Total GAG from control cartilages did not bind to VEGF (panel B) and did not compete with heparin even at a concentration of 1000 μg/mL. Thus, this IC_50_ was defined as a relative low binding affinity of 1%. Conversely, total GAG isolated from OA samples bound to VEGF with an IC_50_ of 136 ± 78 μg/mL (Table 2), showing a significantly higher binding affinity of total GAG from OA samples for VEGF (11.6 ± 1.9%) compared to total GAG from control samples (1 ± 0.1%) (Fig. 3b).

**Table 2:** IC_50_ of total GAG, HS, and the mix of CS and KS for FGF2 and VEGF. IC_50_ were determined in μg/mL and defined as the concentration of tested cartilage GAG able to compete with heparin for 50% of the binding to HBP (FGF2 and VEGF), and used to calculate the % of binding affinity shown in Fig. 3

IC50 (μg/ml)	CT	Femur	Tibial Plateau	Patella	OA
Total GAG					
FGF-2	11 ± 4	88 ± 23	75 ± 23	91 ± 1	84 ± 20
VEGF	NoC	113 ± 76	187 ± 125	106 ± 36	136 ± 78
HS					
FGF-2	34 ± 14	74 ± 31	88 ± 8	110 ± 30	82 ± 25
VEGF	13 ± 2	39 ± 19	45 ± 3	21 ± 2	38 ± 12
CS + KS					
FGF-2	NoC	NoC	NoC	NoC	NoC
VEGF	NoC	NoC	NoC	NoC	NoC

Values correspond to the mean ± SEM for the CT group (*n* = 7) compared to the different parts of OA cartilages: femur (F), tibial plateau (TP), and patella (P) and to all OA cartilage parts (OA, *n* = 11). Each GAG sample was analyzed in duplicate in three independent experiments. *NoC* no competition, *HS* heparan sulfate, *KS* keratan sulfate, *CS* chondroitin sulfate

### HS, CS, and KS cooperate in the cartilage to regulate their binding affinity for growth factors

An ELISA-based competition binding assay was performed with CS/KS (a mix of total GAG devoid of HS) from control and OA cartilages and showed that these GAG were not able to compete with immobilized heparin to bind to FGF2 and VEGF (Table 2). Conversely, HS isolated from control cartilages bound to FGF2 and VEGF with an IC_50_ of 36 ± 14 μg/mL and 13 ± 2 μg/mL, respectively. These values corresponded to a binding affinity of HS for FGF2 and VEGF of 38.1 ± 5.5% and 78.3 ± 5.3%, respectively, compared to the reference values of total GAG from control cartilages (Fig. 3a and b). In OA samples, the binding affinities of HS for FGF2 (11.8 ± 1.4%) and VEGF (36.3 ± 4.5%) were significantly decreased by 3- and 2-folds, respectively, compared to HS control samples (Fig. 3a and b), suggesting that structural changes in HS in OA samples could reduce their affinity for these 2 HBP. Interestingly, the binding affinity for FGF2 in control cartilages was 3 times higher with total GAG (Fig. 3a) (114.7 ± 13.6%) than with isolated HS (38.1 ± 5.5%). This suggested that CS/KS present in total GAG extracts could positively modulate the binding affinity of HS for FGF2. In OA cartilages, total GAG and isolated HS showed similar affinities for FGF2 (12.5 ± 0.9% and 11.8 ± 1.4%, respectively), suggesting that CS/KS present in total GAG extracts were no longer able to increase HS binding affinity for FGF2 in OA cartilages. Regarding VEGF in control cartilages, the binding affinity of HS was significantly higher than that of total GAG (78.3 ± 5.3% and 1 ± 0.1%, respectively), suggesting that CS/KS present in total GAG extracts negatively modulate HS binding affinity for VEGF. In OA samples, the binding affinity of HS for VEGF was only twice as high as that of total GAG (36 ± 4.5% and 11.6 ± 1.9%, respectively), confirming that in the OA context, CS/KS present in total GAG extracts were no longer able to modulate HS binding to VEGF as efficiently as in the control context.

### GAG from OA cartilages induce a catabolic phenotype in murine articular chondrocytes

**Fig. 4:**
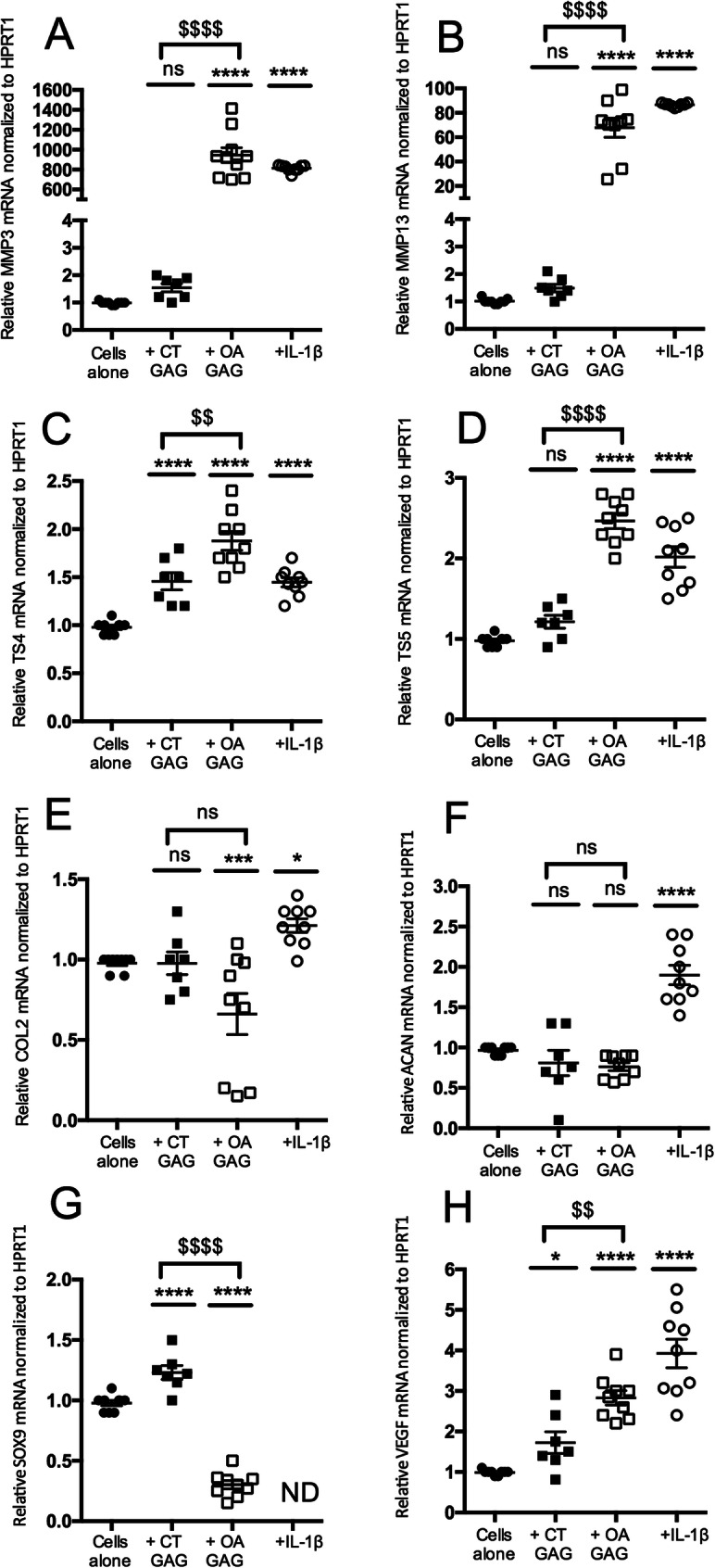
Effect of GAG from cartilages on murine chondrocyte phenotypes. Articular chondrocytes were grown in the absence (cells alone) or in the presence of total GAG (2.5 μg/mL) from the cartilage of control donors (CT GAG) or osteoarthritis patients (OA GAG), or in the presence of IL-1β (1 ng/mL). RQ-PCR analysis of the mRNA expression levels of catabolic markers, MMP3 (**a**), MMP13 (**b**), TS4 (**c**), and TS5 (**d**); anabolic markers, COL2 (**e**), ACAN (**f**) and SOX9 (**g**); and a hypertrophic marker, VEGF (**h**), reported to HPRT1 expression level (housekeeping gene) and compared to the basal condition (cells alone) defined as 1 (100%). Data is presented as the mean of 9 values obtained from 3 independent experiments in CT (*n* = 3 samples) and OA (*n* = 3 samples) cartilages. The statistical significance of the differences was determined using an ordinary one-way ANOVA test followed by pairwise comparisons using the Dunnett test compared to cells alone (*) and by a *t* test between CT GAG and OA GAG ($). Significant *P* values: *< 0.05, **< 0.01, ***< 0.001, ****< 0.0001, ^$$^< 0.01, and ^$$$$^< 0.0001

We investigated whether GAG extracted from OA and control cartilages could affect the anabolic and catabolic phenotypes of murine neonatal articular chondrocytes (Fig. 4). IL-1β, used as a positive control, significantly increased the mRNA expression levels of catabolic markers such as MMP3 (814 ± 11), MMP13 (87 ± 0.4), ADAMTS4 (1.45 ± 0.04), ADAMTS5 (2 ± 0.1), and hypertrophic markers such as VEGF (4 ± 0.3) compared to cells alone. Cells incubated with GAG from control cartilages showed a slight but significant increase in the mRNA levels of TS4 (1.4 ± 0.08), SOX9 (1.2 ± 0.05), and VEGF (1.7 ± 0.26) compared to cells alone. However, cells incubated with GAG from OA cartilages showed significantly increased mRNA levels of all catabolic markers (MMP3 945 ± 74, MMP13 68 ± 7.9, TS4 1.8 ± 0.09, TS5 2.5 ± 0.09) and a hypertrophic marker (VEGF 2.8 ± 0.17) associated with decreased mRNA levels of anabolic markers (COL2 0.6 ± 0.12 and SOX9 0.3 ± 0.03) compared to cells alone. These findings suggested that GAG from cartilages, especially those from OA patients, could inhibit the expression of anabolic markers and induce a catabolic signaling pathway in chondrocytes in vitro.

### Catabolic effects of GAG from OA cartilages are related to their HS species

**Fig. 5:**
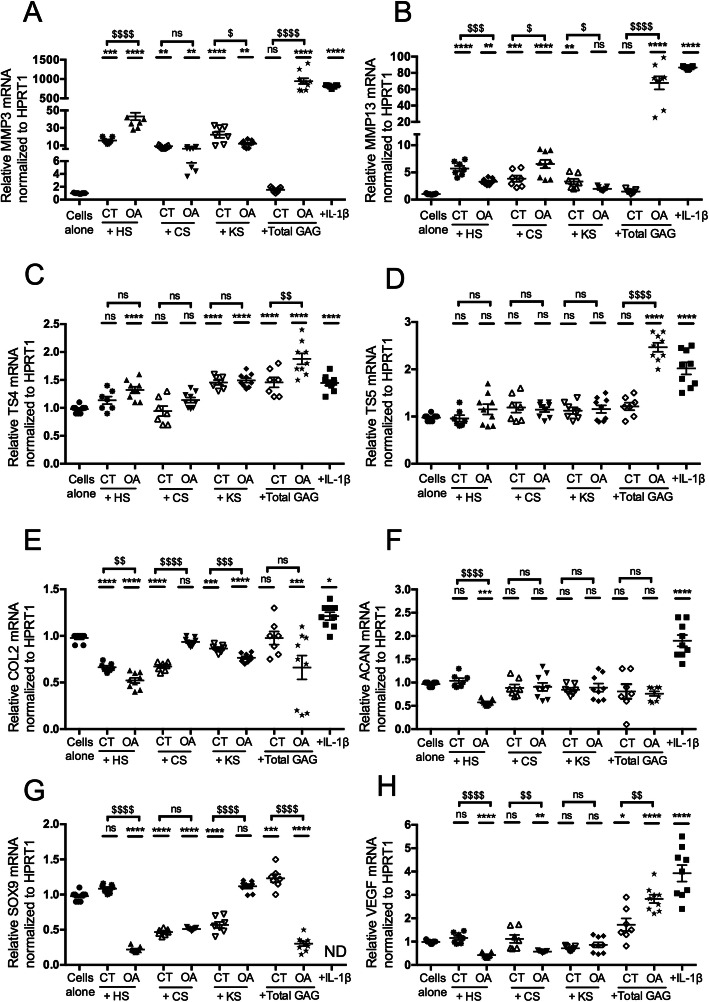
Effect of HS, CS, and KS isolated from cartilages on murine chondrocyte phenotypes. Articular chondrocytes were grown in the absence (cells alone) or in the presence of HS, CS, or KS (2.5 μg/mL) isolated from cartilages of control donors (CT) or osteoarthritis patients (OA), or in the presence of IL-1β (1 ng/mL). RQ-PCR analysis of the mRNA expression levels of catabolic markers, MMP3 (**a**), MMP13 (**b**), TS4 (**c**) and TS5 (**d**); anabolic markers, COL2 (**e**), ACAN (**f**), and SOX9 (**g**); and a hypertrophic marker, VEGF (**h**), reported to HPRT1 expression level (housekeeping gene) and compared to the basal condition (cells alone) defined as 1 (or 100%). Data is presented as the mean of 9 values obtained from 3 independent experiments in CT (*n* = 3 samples) and OA (*n* = 3 samples) cartilages. The statistical significance of the differences was determined using an ordinary one-way ANOVA test followed by pairwise comparisons using the Dunnett test compared to cells alone (*) and by a *t* test between CT GAG and OA GAG ($). Significant *P* values: *< 0.05, **< 0.01, ***< 0.001, ****< 0.0001, ^$^< 0.05, ^$$^< 0.01, ^$$$^< 0.001, ^$$$$^< 0.0001

The gene expression induced by HS, CS, and KS species isolated from cartilages was assessed in primary chondrocyte cultures (Fig. 5). HS from control cartilages significantly increased the mRNA levels of MMP3 (15.6 ± 1.2) and MMP13 (5.7 ± 0.4) and significantly decreased the mRNA levels of COL2 (0.6 ± 0.01) compared to cells alone. HS from OA cartilages showed significantly stronger modulatory effects on 7 out of the 8 markers tested compared to cells alone. Indeed, strong increases in the mRNA levels of MMP3 (43.1 ± 4.2), MMP13 (3.3 ± 0.1), and TS4 (1.3 ± 0.05) and decreases in the mRNA levels of COL2 (0.5 ± 0.02), ACAN (0.5 ± 0.01), SOX9 (0.2 ± 0.01), and VEGF (0.4 ± 0.03) were observed. These findings suggested that cartilaginous HS had catabolic effects that were strongly enhanced in OA samples. Indeed, the difference between HS from control and OA cartilages ($) was significant for 6 out of the 8 markers tested: MMP3, MMP13, COL2, ACAN, SOX9, and VEGF. CS from control cartilages significantly increased the mRNA levels of MMP3 (9.1 ± 0.5) and MMP13 (3.8 ± 0.5) and decreased the mRNA levels of COL2 (0.6 ± 0.01) and SOX9 (0.4 ± 0.01) compared to cells alone. CS from OA cartilages significantly increased the mRNA levels of MMP3 (6.3 ± 0.5) and MMP13 (6.5 ± 0.7) and decreased the mRNA levels of SOX9 (0.5 ± 0.006) and VEGF (0.5 ± 0.01) compared to cells alone. These findings suggested that control cartilaginous CS had catabolic effects on chondrocytes that were maintained in OA samples. Similarly, KS from control cartilages induced a significant increase in the mRNA levels of MMP3 (22.3 ± 3.5), MMP13 (3.3 ± 0.4), and TS4 (1.45 ± 0.04 DDCT) and a decrease in the mRNA levels of COL2 (0.86 ± 0.01) and SOX9 (0.56 ± 0.04) compared to cells alone, suggesting that they could also have a catabolic effect. The same catabolic effect was observed with KS from OA cartilages, with a significant increase in the mRNA levels of MMP3 (12 ± 1.3) and TS4 (1.49 ± 0.03) and a decrease in the mRNA levels of COL2 (0.76 ± 0.01) compared to cells alone. The difference between KS from control and OA samples was highly significant for the mRNA levels of COL2 and SOX9. CS and KS from OA samples were no longer able to inhibit the mRNA levels of these two anabolic markers. These findings suggested that CS, KS, and HS were individually able to disrupt the balance between anabolism and catabolism in chondrocytes but that the HS species had the strongest effect.

## Discussion

The role of HSPG in OA has been barely investigated, in particular changes in their functional capacities according to the degree of sulfation of HS chains. Our GAG extraction protocol allowed isolating sufficient amounts of HS from cartilages for studying their structure and functional effects, including their binding affinity for HBP. We found a 2-fold decrease in HS levels in OA cartilages compared to controls, in accordance with previous works that have shown a lower decrease in HS probably due to a lower yield of HS extraction [7]. Changes also affected the composition of other GAG.

We showed that the levels of monosulfated CS disaccharides were increased in OA cartilages. This finding is in line with a report showing an increase in monosulfated CS levels (GalNAc 6-O-sulfation) in OA and changes in the CS4/CS6 ratio [22]. This could be related to changes in aggrecan composition, which in turn could influence chondrocyte metabolism and properties, resulting in OA but the underlying mechanisms are still poorly understood [23]. Monosulfated disaccharides were the main HS forms observed in human control cartilages, and this fraction was increased in OA, together with the appearance of disulfated disaccharides. This could be due to the presence of neosynthesized HS with N-sulfation, due to the activity of the sulfotransferase Ndst which determines high sulfation regions in the HS chain. Parra et al. have shown a very high degree of sulfation, similar to that of heparin, of minor HS present in growth plates and mature articular cartilages in young rabbits using a NMR structural analysis [21, 22]. Such highly sulfated HS could be produced as a functional response to the need of a growing tissue with strong chondrocyte proliferation. Accordingly, Chanalaris et al. have shown a significant increase in 6-O-sulfation in HS disaccharides from human OA samples compared to controls [7]. Thus, it could be assumed that the sulfation pattern of HS could vary according to species, age, cartilage type, and cell metabolism.

Compared to other published studies assessing changes in HS levels and sulfation pattern in OA, the main original result of our work is that we observed changes in the binding affinities of HS, CS, and KS isolated from OA samples for HBP and a change in the balance between anabolism and catabolism in chondrocytes.

It is likely that, in the in vitro cell system used, the plated chondrocytes expressed cell surface and pericellular HS proteoglycans that bound to HS ligands present in the culture medium, with a maintained balance between the anabolic and catabolic activities. Thus, the increased catabolic effects observed when adding HS from OA cartilages could be explained by different biological effects: (i) a direct effect on the binding and activity of catabolic factors present in the culture medium and/or secreted by chondrocytes and that will be strongly induced to interact with their receptors, inducing catabolic phenotypes and (ii) an indirect or competitive effect, since exogenous GAG could displace anabolic growth factors from the cell layer, preventing their interaction with their receptors. Thus, the increased catabolic effects observed in the presence of HS from OA cartilages could be due to a higher affinity for all types (anabolic or catabolic) of growth factors.

Interestingly, we showed that total GAG from control cartilages were able to bind to FGF2, but mainly due to the binding affinity of HS for this HBP, which was decreased in OA cartilages. FGF2 is endogenously produced by chondrocytes and sequestered by perlecan, a matrix HSPG, in the ECM of the articular cartilage [24, 25]. When the cartilage is damaged, FGF2 is released from perlecan HS chains [26] to subsequently activate the ERK signaling pathway [27]. This has been associated with catabolic effects in human articular chondrocytes via an upregulation of matrix degrading enzymes, the inhibition of ECM accumulation, an increased PG synthesis, and the clustering of cells characteristic of OA [28]. We showed here that altered HS levels and sulfation patterns in human OA cartilages were associated with a decreased binding affinity of HS for FGF2. We hypothesized that this lower binding affinity of HS chains for FGF2 could be associated with a decrease in its sequestration by perlecan in the ECM and thus with an increase in FGF2 bioavailability and catabolic activity on chondrocytes.

We observed increased levels of monosulfated and disulfated disaccharides forms of HS from OA cartilages that could be due to a first increased level of N-sulfation pattern. Recently, Severmann et al. have analyzed the role of HS levels and sulfation patterns in OA induced in transgenic mice carrying a chondrocyte-specific loss-of-function allele of Ext1 and of Ndst1 [29]. Mouse strains with reduced HS levels and sulfated HS showed reduced OA scores, suggesting that “highly” sulfated HS could regulate cartilage degeneration through an effect on the protease activity. FGF2 binds preferentially to HS containing iduronate-2-O-sulfation and glucosamine-N-sulfates, whereas 6-O-sulfation is essential for the receptor engagement in the trimolecular FGF2/FGFR/HS signaling complex and its mitogenic activity [30–32]. The 6-O-sulfation levels clearly regulate FGF2-induced receptor phosphorylation, FGFR1 internalization, and downstream FGF2-dependent endothelial phenotypes in vitro and in vivo [33].

We showed that while total GAG from control cartilages did not bind to VEGF, total GAG from OA cartilages were able to bind to it and this still mainly due to the binding affinity of HS for this HBP. Interestingly, VEGF expression is increased in OA cartilages and correlates with the severity of the disease. Accordingly, VEGF is a procatabolic and proangiogenic factor in OA that induces MMP13 expression, osteophyte formation, and differentiation towards a hypertrophic state [21]. In animal models of OA, VEGF blockade using monoclonal antibodies or in KO mice decreased OA severity, while an intra-articular injection of VEGF worsened cartilage damage [26, 27]. Thus, our data suggests that the increased binding affinity of total GAG from OA cartilages for VEGF could play a role in various catabolic processes.

Key structural features of highly sulfated HS domains mediate the specific binding of the VEGF-165 dimer to its receptors: the carboxylate groups and 2-O-, 6-O-, and N-sulfation of HS contribute to the strength of VEGF-165 interactions. However, N-sulfates and 6-O-sulfates appear to be particularly important for VEGF-165 binding [34]. While a high content of 2-O-sulfate groups is not required for the specific interaction with VEGF-165, it is essential for its mitogenic activity [35]. Moreover, HS structures with reduced 6-O-sulfation negatively affect VEGF-165-dependent endothelial phenotypes in vitro and in vivo, confirming that the 6-0-sulfation pattern is important for the biological activity [33].

As described in the literature, N-sulfated HS and/or sulfated 6-O-HS are increased during OA leading to enhanced binding affinity of HS to FGF2 and VEGF. However, our in vitro binding assay on HS isolated from cartilage indicates that increased sulfation of HS in OA is associated with reduced binding of HS to FGF2 and VEGF. This suggests that much more complicated sulfation patterns of HS chains are involved, a process that is probably timely regulated. Moreover, they could have different modulatory effects on cells depending on the successive interactions with their receptors when CS and KS are present.

In this context, newly produced sulfated HS, as shown by the OA-related generation of disulfated disaccharides, could require changes in the expression pattern of the complex network of enzymes involved in HS biosynthesis such as anabolic glycosyl transferases (EXTs and EXTLs), epimerases and sulfotransferases (NDSTs and HSTSs), as well as catabolic heparanase and HS 6-O-endosulfatases (Sulfs) [36]. Increased 6-O-sulfation levels in OA have recently been correlated with an increased expression of HS6ST1, a 6-O-sulfotransferase, and GLCE, an epimerase promoting 6-O-sulfation, suggesting that changes in 6-O-sulfation could have an impact on a key signaling pathway in the cartilage [7]. Moreover, the endosulfatases, Sulf1 and Sulf2, are overexpressed in human OA tissues [37, 38] and spontaneous cartilage degeneration and surgically-induced OA have been shown to be significantly more severe in Sulf1^−/−^ and Sulf2^−/−^ mice compared to wild-type mice [39]. Accordingly, an intra-articular injection of Sulf1 in a mouse model of OA prevented cartilage degeneration [40]. This suggests that 6-O-HS desulfation could have a protective effect in OA. Our results are in accordance with these findings demonstrating the functional relevance of oversulfated HS, with an impaired binding to FGF2 and VEGF and a resulting catabolic phenotype in chondrocytes in OA.

In this work, we focused on developing a protocol for HS isolation, while ensuring efficient digestions with chondroitinase and keratinase. No purification step of the disaccharides was performed because previous data from the literature has shown the efficacy of chondroitinase ABC digestion in reducing the functional activity of various CS preparations. It has been shown that digested CS-E and CS-H are no longer able to bind to heparin-binding growth factors (HBGF) such as FGF2, FGF1, FGF18, or PTN; inhibit the binding of full-length CS to HBGF; and induce neurite outgrowth of embryonic rat hippocampal neurons [41]. This phenomenon has been shown to be size dependent with CS disaccharides losing all their binding and biological activities [42]. This suggests that CS disaccharides, although they are much more concentrated than HS, will not have any effect on the binding of HS to protein ligands.

Finally, our binding experiments support an important regulatory role of the CS/KS species in the binding properties of GAG extracted from cartilages, reflecting their modulatory effects on HS affinity for FGF2 and VEGF. In control cartilages, HS were able to bind to FGF2, but with total GAG extracts containing a mix of CS and KS, this binding affinity was increased, perhaps to allow a better retention of FGF2 in the matrix. Similarly, HS from control cartilages bound to VEGF with a strong affinity, but the binding of the mix of CS and KS in total GAG extracts was completely suppressed. Although differences observed in an in vitro biochemical binding test are less biologically significant than in vivo findings, our results suggest that HS, CS, and KS interactions are regulated by a complex interplay that could depend on the HBP involved and on the proteoglycans carrying the GAG chains. Our data revealed that in OA samples, together with the decreased binding affinity of HS for FGF2 and VEGF, the modulatory properties of CS/KS were lost or decreased. The mix of CS and KS was no longer able to increase HS binding to FGF2 and to decrease HS binding to VEGF. This could also be due to complex structural changes in and/or an altered sulfation of CS and KS chains.

Such an assumption should also take into account the real location of HS, CS, and KS chains on PG in the cartilage. While, in the cartilage, HS are mainly located in the pericellular region, most CS/KS on aggrecan are located in the inter-territorial zone and are largely excluded from the pericellular region. However, this does not exclude the fact that some pericellular HSPG, such as perlecan or syndecan, could be substituted for CS or KS chains associated with HS chains. Mammalian perlecan is predominantly substituted for HS chains but it may also be substituted for CS, dermatan sulfate (DS), HS/CS hybrid, and CS/DS chains at the main GAG attachment sites on domains I [43, 44] and V [45]. Syndecans were the first hybrid-type PG described to carry both HS and CS chains. Syndecans from different cell types are found as discrete isoforms with variability in the type, number, and size of GAG chains attached, resulting in various potential interactions [46]. The role of HS chains is to attract and present different proteins at the cell surface, whereas the role of CS (and the related DS) chains, that bind to matrix proteins [47] and soluble molecules [48], would be to change the interactions between syndecans, their HS chains, and other proteins, as shown for midkine and pleiotrophin [49, 50]. We could therefore propose a molecular mechanism in which the CS/KS chains viscinity with HS chains on the same core protein and the associated polyvalent chain arrangement will affect and stabilize the direct interactions between HS chains and HBP, even if here, the CS and KS chains from cartilages did not directly interact with the tested HBP. Our results suggest a new role of CS and KS that could regulate the availability of HS functional chains for their target HBP. The complex interactions between the various sulfation patterns of the mix of HS, CS, and KS remain to be elucidated.

## Conclusions

In this study, we showed that GAG from human OA cartilages were able to change the catabolic/anabolic balance of normal chondrocytes and, more surprisingly, that this effect was mainly due to HS functional properties in a cartilage matrix enriched in CS and KS. It remains to be clarified whether a particular PG core protein is carrying the deleterious HS chains or if these chains are present in all HSPG expressed in OA articular cartilages, and how the mix of HS, KS, and CS interplay to promote OA during aging.

## Data Availability

The datasets used and/or analyzed in this study can be provided by the corresponding author on reasonable request.

## Abbreviations

ADAMTS: Disintegrin and metalloproteinase with thrombospondin
cDNA: Complementary DNA
COL2: Type 2 collagen
CS: Chondroitin sulfate
CT: Control
DMMB: Dimethyl-methylene blue
ECM: Extracellular matrix
ELISA: Enzyme-linked immunosorbent assay
ERK: Extracellular signal-regulated kinase
EXTLs: Exostosin-like
EXTs: Exostosin
F: Femur
FGF: Fibroblast growth factor
GAG: Glycosaminoglycans
GalNAc: N-Acetylgalactosamine
GLCE: Glucuronyl C5-epimerase
HA: Hyaluronic acid
HBP: Heparin-binding protein
HPLC: High-performance liquid chromatography
HPRT: Hypoxanthine-guanine phosphoribosyltransferase
HS: Heparan sulfate
HSPG: Heparan sulfate proteoglycans
HSTS: Heparan sulfate sulfotransferases
IC_50_: Half maximal inhibitory concentration
IL-1β: Interleukin-1 beta
KO: Knockout
KS: Keratin sulfate
MMP: Matrix metalloproteinases
NDST: N-deacetylase/N-sulfotransferases
NMR: Nuclear magnetic resonance
OA: Osteoarthritis
P: Patella
PG: Proteoglycan
RNA: Ribonucleic acid
RQ-PCR: Quantitative polymerase chain reaction
SOX9: Transcription factor SOX-9
TP: Tibia plateau
VEGF: Vascular endothelial growth factor
